# When the time is ripe

**DOI:** 10.7554/eLife.00958

**Published:** 2013-06-25

**Authors:** Siobhan M Brady

**Affiliations:** 1 **Siobhan M Brady** is at the Department of Plant Biology and Genome Center, University of California, Davis, California, United Statessbrady@ucdavis.edu

**Keywords:** transcriptional regulation, temporal modulation, network, ethylene, hormone, plant biology, Arabidopsis

## Abstract

The diverse effects of the plant hormone ethylene on development and growth are shaped by the actions of a master regulator of transcription, EIN3.

**Related research article** Chang KN, Zhong S, Weirach MT, Hon G, Pelizzola M, Li H, Huang S-sC, Schmitz RJ, Urich MA, Kuo D, Nery JR, Qiao H, Yang A, Jamali A, Chen H, Ideker T, Ren B, Bar-Joseph Z, Hughes TR, Ecker JR. 2013. Temporal transcriptional response to ethylene gas drives growth hormone cross-regulation in *Arabidopsis*. *eLife* **2**:e00675. doi: 10.7554/eLife.00675

**Image** Mutants of EIN3 targets (lower panels) show impaired flowering and leaf morphology

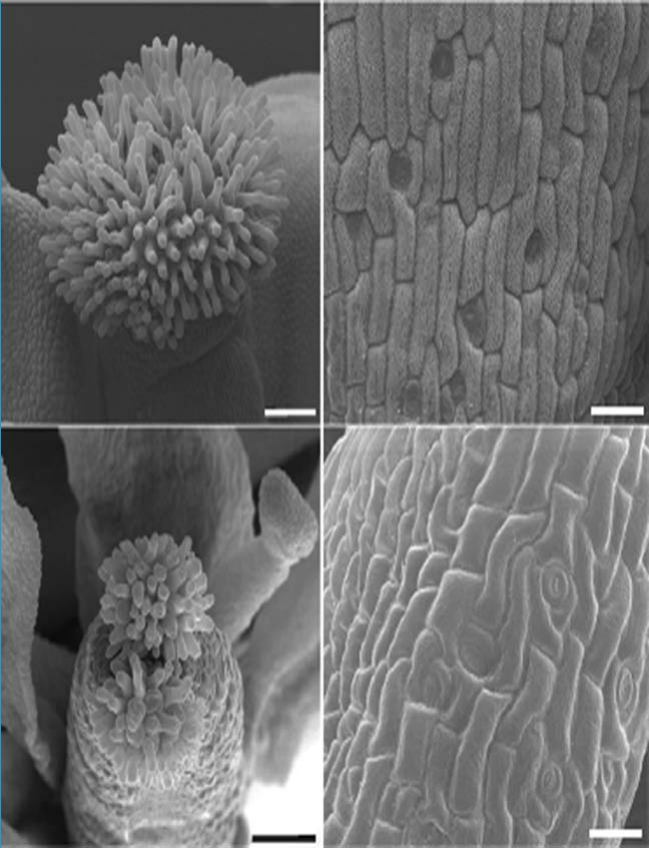

Recall the moment you bit into a tomato that looked so ripe and red on the outside and that was sure to be juicy and delicious! Immediately your mouth was filled with something that was neither juicy nor delicious. These bland tomatoes were likely harvested before they were ripe so that they could be transported, and were later gassed with ethylene to accelerate the ripening process. It has been known for more than 100 years that ethylene is a hormone that controls many aspects of the growth of plants, including how fruits ripen. A detailed mechanistic understanding of how plants detect ethylene and use it to coordinate biological processes is therefore of great importance in agriculture. Genetic analysis has identified a number of key components of the ethylene signalling pathway, including receptors and their downstream transcription factors. However, the technology needed to identify the targets of these transcription factors and the dynamics of these regulatory events in plants has only recently become available. Now, in *eLife*, Joseph Ecker at the Salk Institute for Biological Studies and co-workers use such technology to explore how plants respond to ethylene (Chang et al., 2013).

A transcription factor known as Ethylene Insensitive 3 (EIN3) is believed to act as a master transcriptional regulator of the ethylene response, and to coordinate the expression of downstream genes that direct ethylene-mediated growth. Transcription factors can be made up of multiple protein domains, and these might include a DNA binding domain (which recognizes specific target DNA sequences) or a domain that interacts with the transcriptional machinery to regulate the expression of genes**.** By combining these domains in different ways, transcription factors can act in a modular fashion. The targets of the DNA binding domain can be identified using a technique called ChIP-seq, in which chromatin immunoprecipitation (ChIP) of a transcription factor is coupled to sequencing (-seq) of its target DNA. However, when a transcription factor binds to DNA, it does not necessarily regulate the expression of that gene, in part due to the modular nature of transcription factors. In fact, experiments using ChIP-seq technology in humans have shown that transcription factors regulate the expression of only 1–10% of the targets that they bind to (Farnham, 2009). This may be because these types of studies often capture only a single time point, whereas transcriptional responses are dynamic and change over time.

Ecker and colleagues—including Katherine Chang as first author—explored this issue by exposing the model plant *Arabidopsis thaliana* to ethylene and then using ChIP-seq to identify sequences bound by EIN3. To determine which of those sequences EIN3 regulates, they profiled whole genome expression and EIN3 target sequences at an unprecedented seven time points after ethylene application. This revealed that 30% of the direct targets of EIN3 had their expression regulated in response to ethylene. Moreover, the transcriptional response could be catalogued into four distinct waves of gene expression. The first wave occurred 15 minutes after ethylene application and was extremely variable and ‘noisy’. After thirty minutes, in the second wave, EIN3 began to bind to and regulate the expression of its targets more frequently, and subsequent waves of gene expression were less variable.

One potential mechanism for the reduction of transcriptional noise at later time points is through EIN3-dependent feedback. EIN3 binds to and regulates the expression of multiple genes that serve as negative regulators of the ethylene response, including Ethylene Triple Response1 (ETR1) and Constitutive Triple Response1 (CTR1) (Figure 1A). This can dampen the ethylene response and provide a mechanism for system homeostasis.

**Figure 1.**
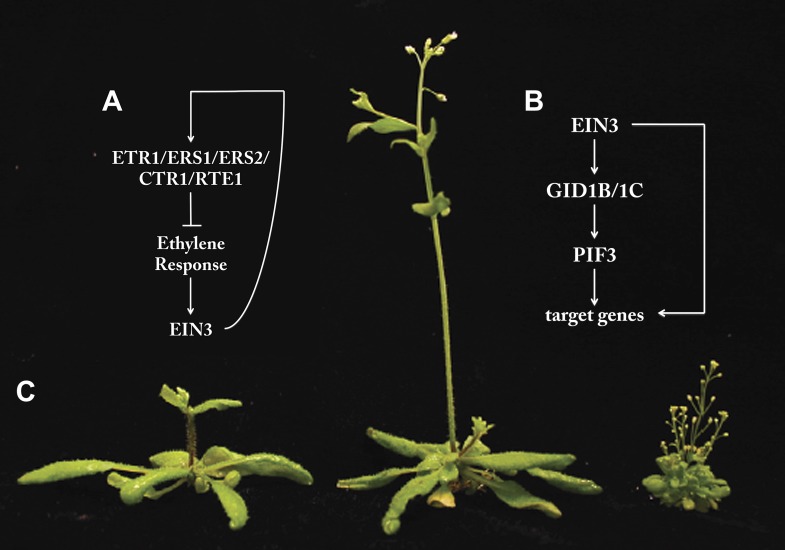
The transcription factor EIN3 is a master regulator of transcription, and acts through multiple feedback and feedforward regulatory mechanisms. (**A**) The plant hormone ethylene increases the activity of EIN3. However, EIN3 in turn promotes the expression of proteins that inhibit the response to ethylene via a feedback loop. (**B**) EIN3 acts both directly and indirectly to increase the expression of its target genes. The indirect feedforward loop involves increased expression of receptors for another plant hormone, gibberellic acid (GID1B/1C). These in turn activate the transcription factor PIF3, which increases expression of EIN3 target genes. (**C**) The background image shows wildtype *Arabidopsis thaliana* (left) alongside plants with mutations in one (middle) or four (right) EIN3 target genes (adapted from Figure 4F from Ecker et al., 2013).

Depending on the time point sampled, EIN3-bound targets made up approximately 20–30% of genes that showed altered transcription in response to ethylene. However, the results of Chang et al. demonstrate that EIN3 also binds to and regulates the expression of transcription factors and genes within other hormone-mediated signalling pathways. Altogether, approximately 65% of the genes that were directly regulated by EIN3 were hormone-related. Most of these were involved in ethylene signalling, but some were implicated in the response to multiple hormones. The result is a complex transcriptional cascade, in which EIN3 binds to and regulates the expression of a multitude of transcription factors and hormone signalling genes—which in turn go on to influence other protein-DNA and protein-protein interactions—within a hormone co-regulation network. The EIN3 gene regulatory system is thus elaborate and interconnected, consistent with the role of EIN3 as a master transcriptional regulator.

One elegant example of the interconnection of these downstream responses is a feedforward loop that integrates gibberellic acid (another plant hormone) and signalling in response to light. EIN3 binds to and regulates the expression of gibberellic acid receptors, GID1B and GID1C. Activation of these receptors regulates the activation of a transcription factor called PIF3 (Feng et al., 2008; de Lucas et al., 2008), and genes that are targeted by EIN3 also have PIF3 binding sites in their promoters (Figure 1B). Feedforward loops of this type can buffer regulatory systems from stochastic noise (Shen-Orr et al., 2002).

In an effort to identify additional feedforward or feedback loops, Chang et al. used protein binding microarrays. This technology has previously been used in mice and in the nematode worm, *C. elegans*, to identify the specific DNA sequences within promoters that are targeted by transcription factors (Badis et al., 2009; Grove et al., 2009). In the current study, Chang et al. identified such sequences for 12 transcription factors that are targeted by EIN3 in response to ethylene. This predicted secondary response to ethylene adds additional complexity to the EIN3-mediated gene regulatory network.

How relevant are EIN3-target genes in controlling ethylene-mediated growth and development? Since ethylene regulates a plethora of processes, one might predict that disruption of target genes would seriously impair plant growth. To test this hypothesis, Chang et al. knocked down the expression of three EIN3 target genes from the HOOKLESS family, including one that is known to integrate ethylene, light and sugar signalling responses as well as those of two other plant hormones, auxin and brassinosteroid (Hou et al., 1993; Li et al., 2004; De Grauwe et al., 2005; Ohto et al., 2006). Knockdown of this family—and hence a key component of the EIN3-dependent gene regulatory network—did indeed result in severe developmental defects (Figure 1C). Thus, incorporation of cutting-edge genomics technology, temporal dynamics and systems level analysis has provided a beautiful example of how a hormone is perceived and its signal is transcriptionally transduced to coordinate growth and development. Now, we need to wait to see how this research can be translated into making our tomatoes taste better!
